# Supplementary material for: Can Crude Oil Exploration Influence the Phytochemicals and Bioactivity of Medicinal Plants? A Case of Nigerian Vernonia amygdalina and Ocimum gratissimum
Source: Molecules. 2022 Nov 30;27(23):8372. doi: 10.3390/molecules27238372 (PMC9740812; doi:10.3390/molecules27238372)
Supplement: Supplementary file 1 [file molecules-27-08372-s001.zip › molecules-2010861-supplementary revised.pdf]

Article

Can Crude Oil Exploration Influence the Phytochemicals and Bioactivity of Medicinal Plants? A Case of Nigerian *Vernonia amygdalina* and *Ocimum gratissimum*

Oluwatofunmilayo A. Diyaolu [1,*], Emmanuel T. Oluwabusola [1], Alfred F. Attah [2], Eric O. Olori [3], Adeshola A. Fagbemi [4], Gagan Preet[1], Sylvia Soldatou[1], Jones O. Moody [5], Marcel Jaspars [1], and Rainer Ebel [1,*]

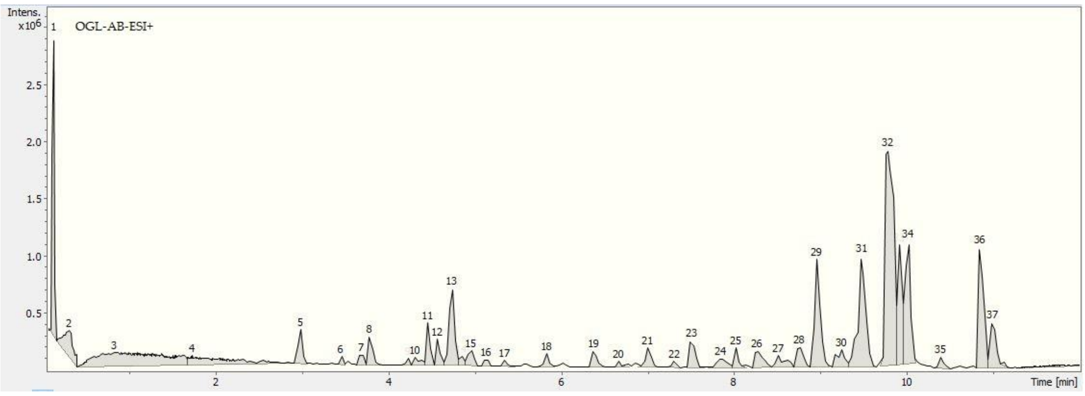

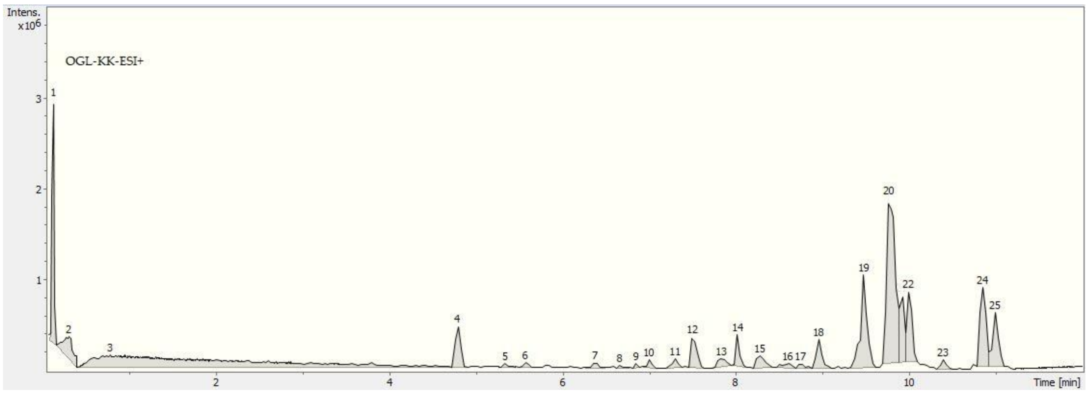

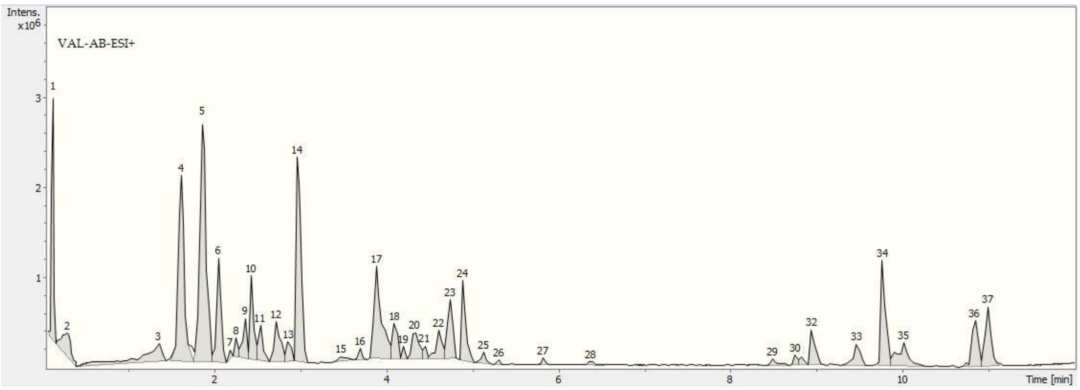

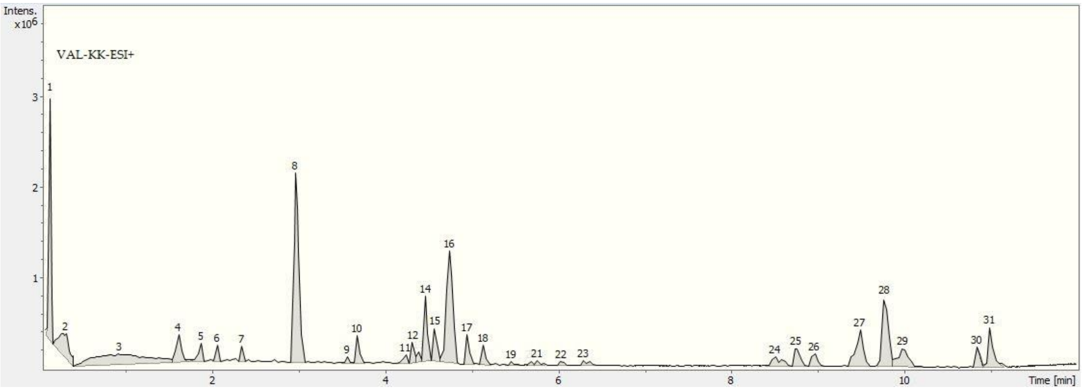

Figure S1. The representative base peak chromatograms (BPC) of VAL and OGL in Electrospray Ionisation (ESI^+) positive mode

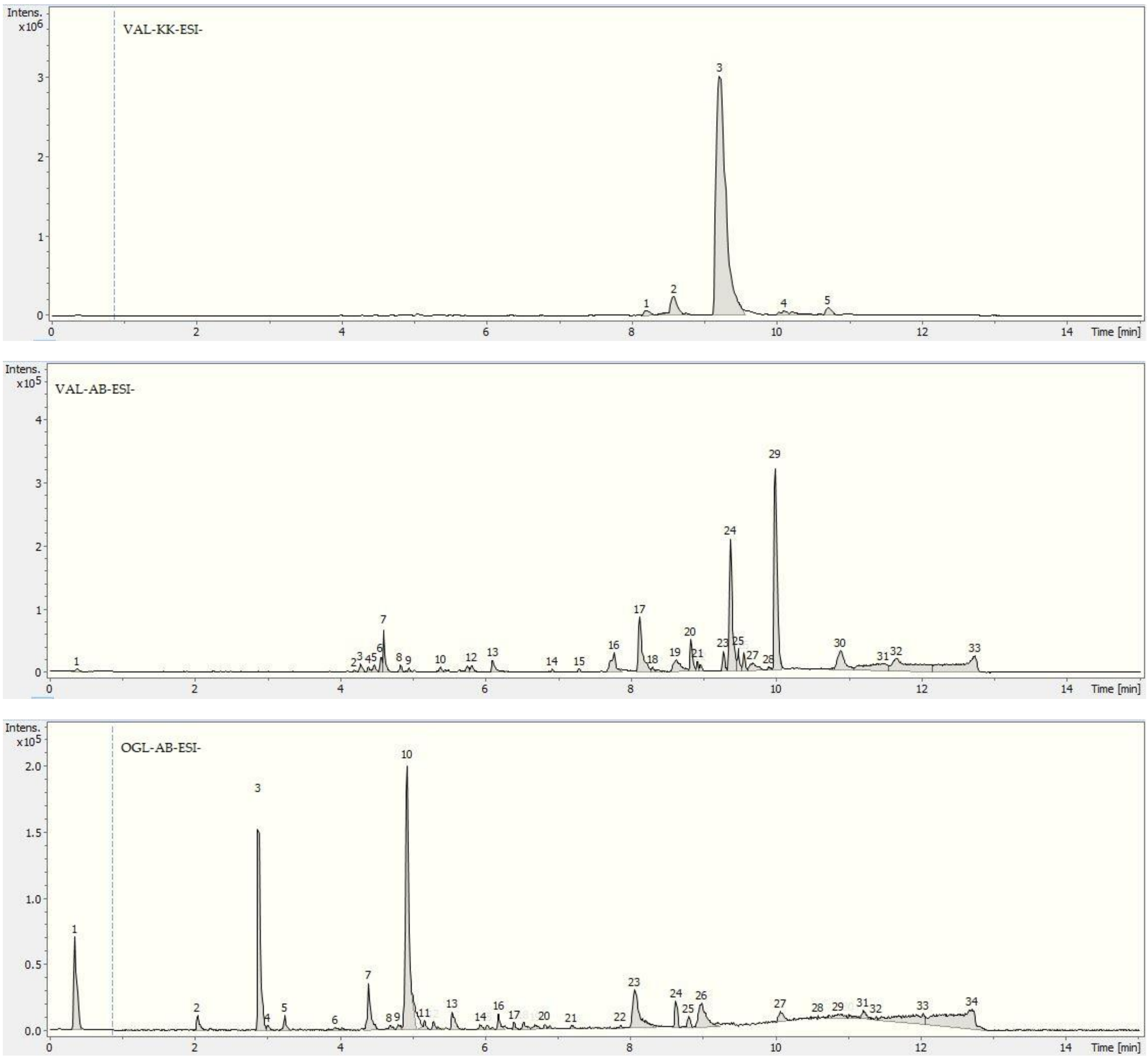

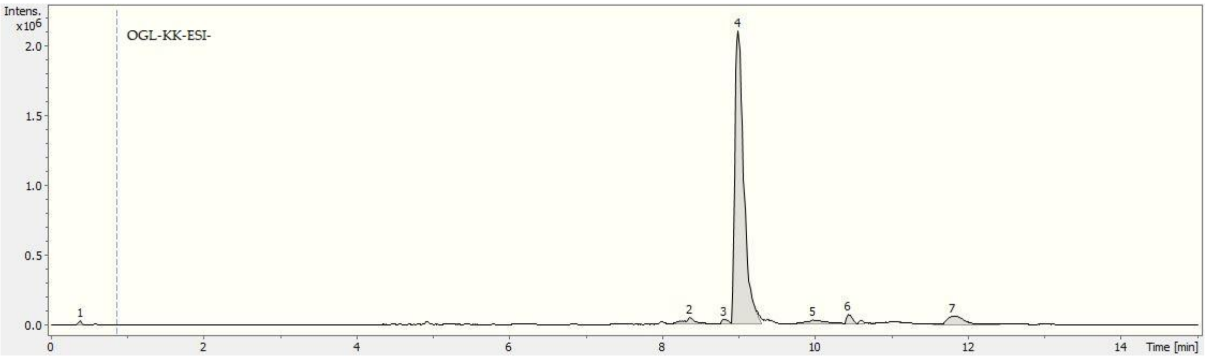

Figure S2. The representative base peak chromatograms (BPC) of VAL and OGL in Electrospray Ionisation (ESI^-) negative mode

Table S1: NMR data for vernodalol in CD_3OD (400 MHz)

Position	δ_C, mult	δ_H, mult (*J* in Hz)	COSY	HMBC (H→C)
1	142.1 CH	5.78 dd (17.7, 11.4)	2	2,5,10,14
2	115.6 CH_2	A 5.26 d (17.7)<br>B 5.22 d (11.1)	1	5,10,14
3	166.6			
4	134.1			
5	51.9 CH	2.57 dd (10.7, 1.7)	6	2,6,8,9,10,12,14,15
6	69.7 CH	4.06 dd (11.0, 10.3)	5,7	15
7	54.5 CH	2.75 dd (10.6, 11.4)	6,8	5,8,9,11,12,13
8	70.4 CH	5.39 dt (4.9, 11.4)	7,9	1′,5,7,13
9	37.5 CH_2	A 2.02 dd<br>B 1.68 t	8	1,2,7,8,10,14
10	40.5			
11	138.3			
12	168.1			
13	129.7 CH_2	A 6.50 d (1.6)<br>B 5.47 d (1.6)		12
14	71.9 CH_2	A 4.70 d (11.9)<br>B 4.44 d (2.4, 12.0)	14	1,5,9
15	134.2	A 6.54 d (1.6)		3

	CH_2	B 5.77 d (1.6)		
16	52.0 CH_3	3.76 s		
1′	166.1			
2′	140.4			
4′	61.4 CH_2	4.18 m	3′	1′,2,3′
3′	124.0<br>CH_2	A 6.19 d (1.3)<br>B 5.87 d (1.7)	4′	1′,2′,4′

δ- Chemical shift

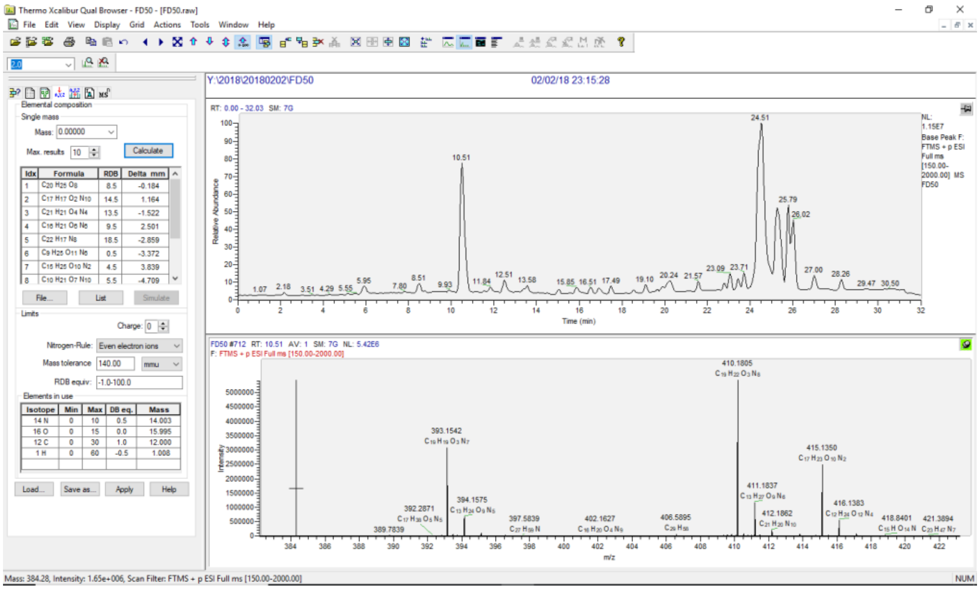

Figure S3: MS data for vernodalol

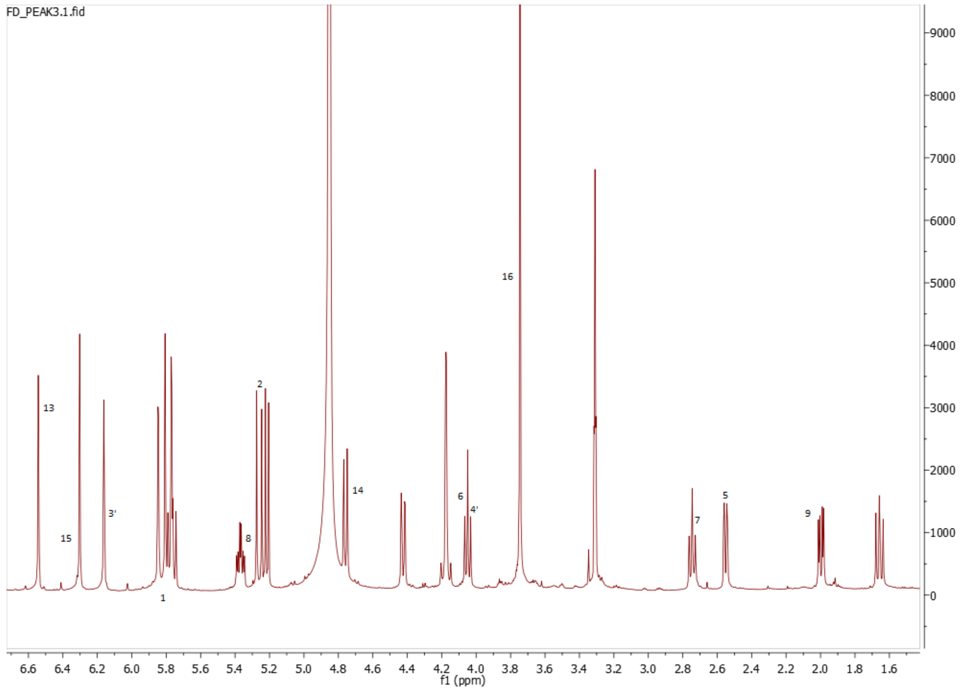

Figure S4: ^{1}H NMR spectral of vernodalol

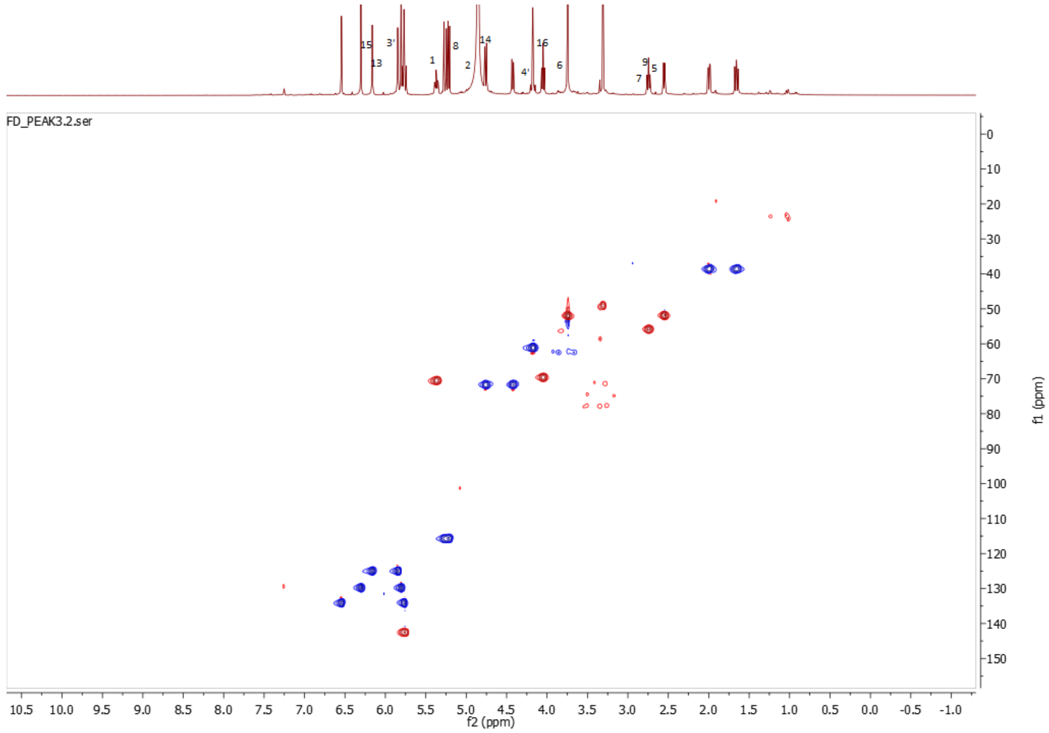

Figure S5: HSQC spectral of vernodalol

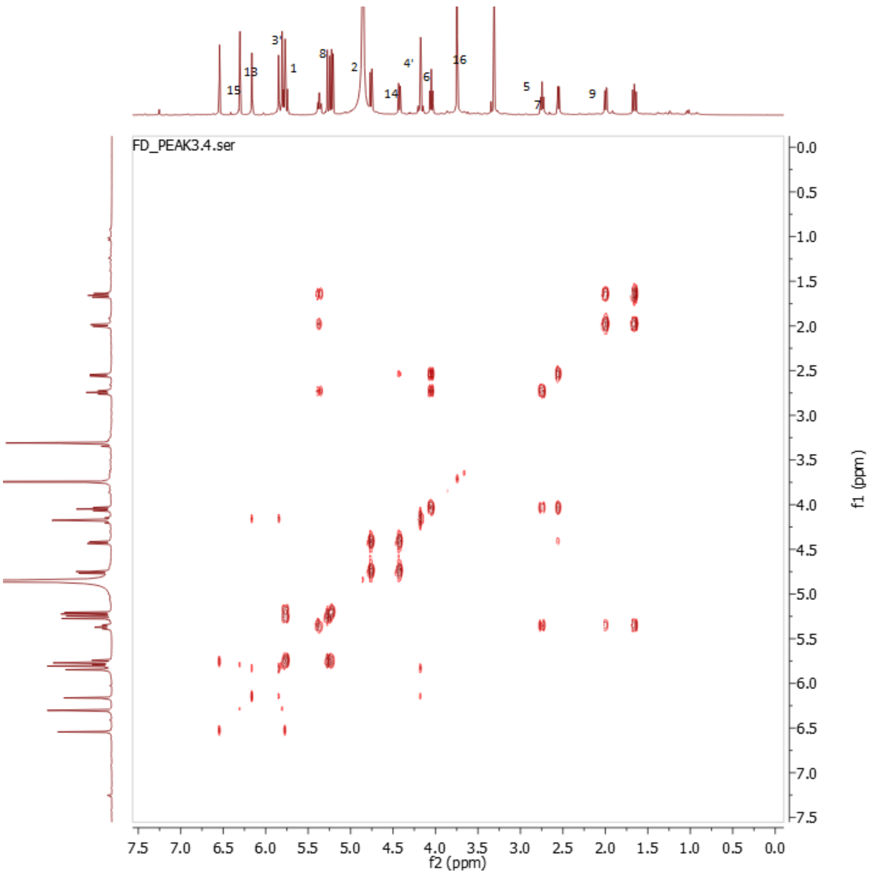

Figure S6: COSY spectral of vernodalol

Table S2: NMR data for lasiopulide in CD_3OD (400 MHz)

Position	δ_C , mult	δ_H, mult (*J* in Hz)	COSY	HMBC (H→C)
1	142.7 CH	5.73 dd (17.7, 11.4)	2	2,5,14
2	115.1<br>CH_2	A 5.21 d (17.71)<br>B 5.22 d (11.14)	1	14
3	166.4			
4	133.9			
5	51.9 CH	2.45 dd (10.74, 1.74)	6	2,6,8,9,12,14,15
6	66.8 CH	4.06 dd (10.30)	5,7	15
7	59.3 CH	2.40 dd (10.57)	6,8	5,8,9,12,13
8	69.9CH	5.39 td (11.40, 4.91)	7,9	5,7,13
9	40.0<br>CH_2	A 2.02 dd<br>B 1.68 t	8	1,2,7,8,14

10	42.4			
11	138.8			
12	168.0			
13	129.5<br>CH_2	A 6.37 d (1.58)<br>B 5.79 d (1.58)		
14	72.1<br>CH_2	A 4.70 d (11.94)<br>B 4.44 d (11.99, 2.37)	14	1,5,9
15	133.8<br>CH_2	A 6.52 d (1.58)<br>B 5.74d (1.58)		3
16	51.8 CH_3	3.75 s		

δ- Chemical shift

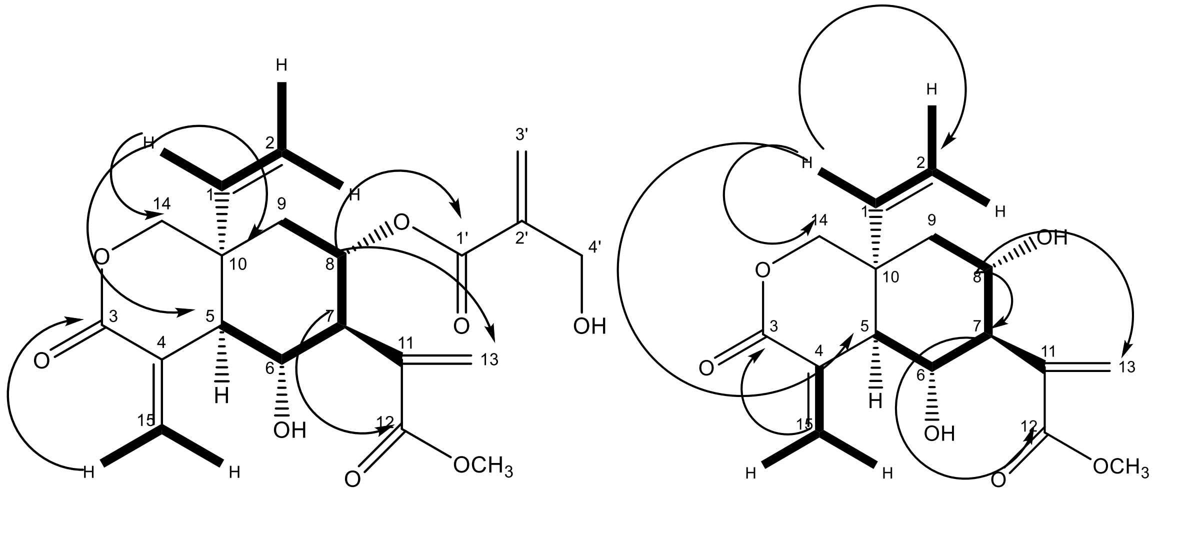

Figure S7: Structures and 2D NMR correlations of vernodalol and lasiopulide
(**bold**: COSY, arrows: HMBC, from H to C)

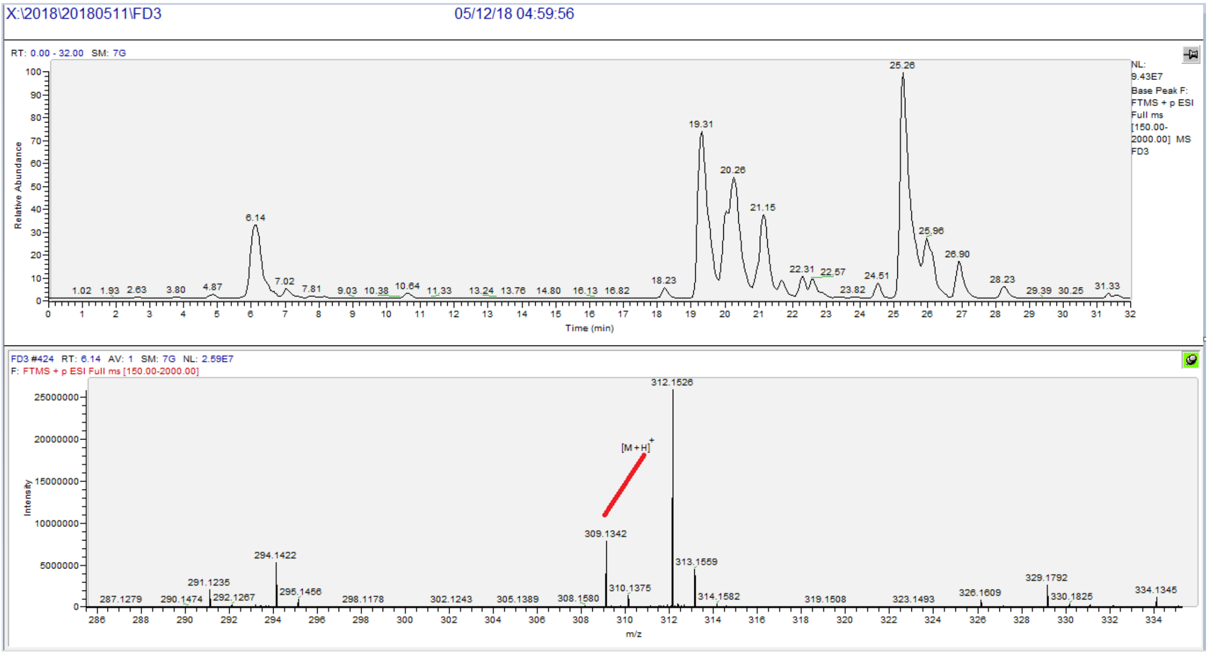

Figure S8: MS of lasiopulide

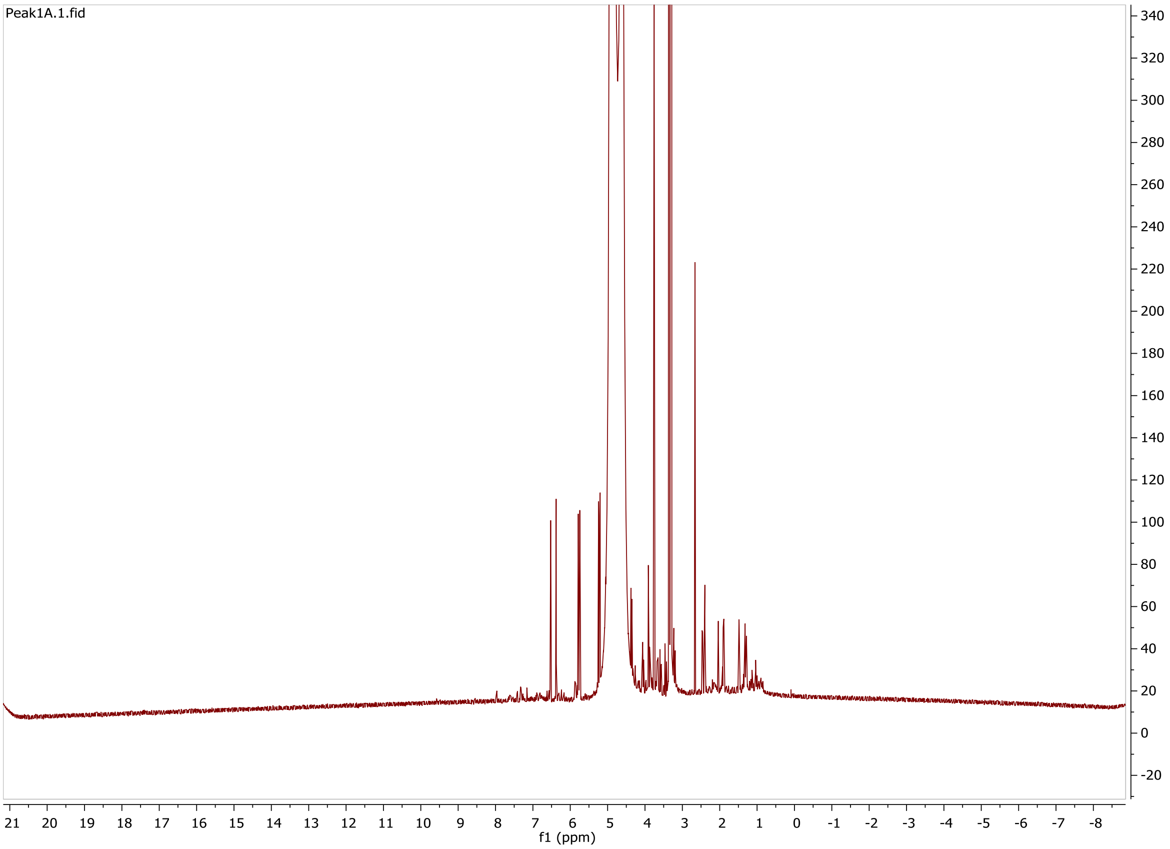

Figure S9: ^{1}HNMR of lasiopulide

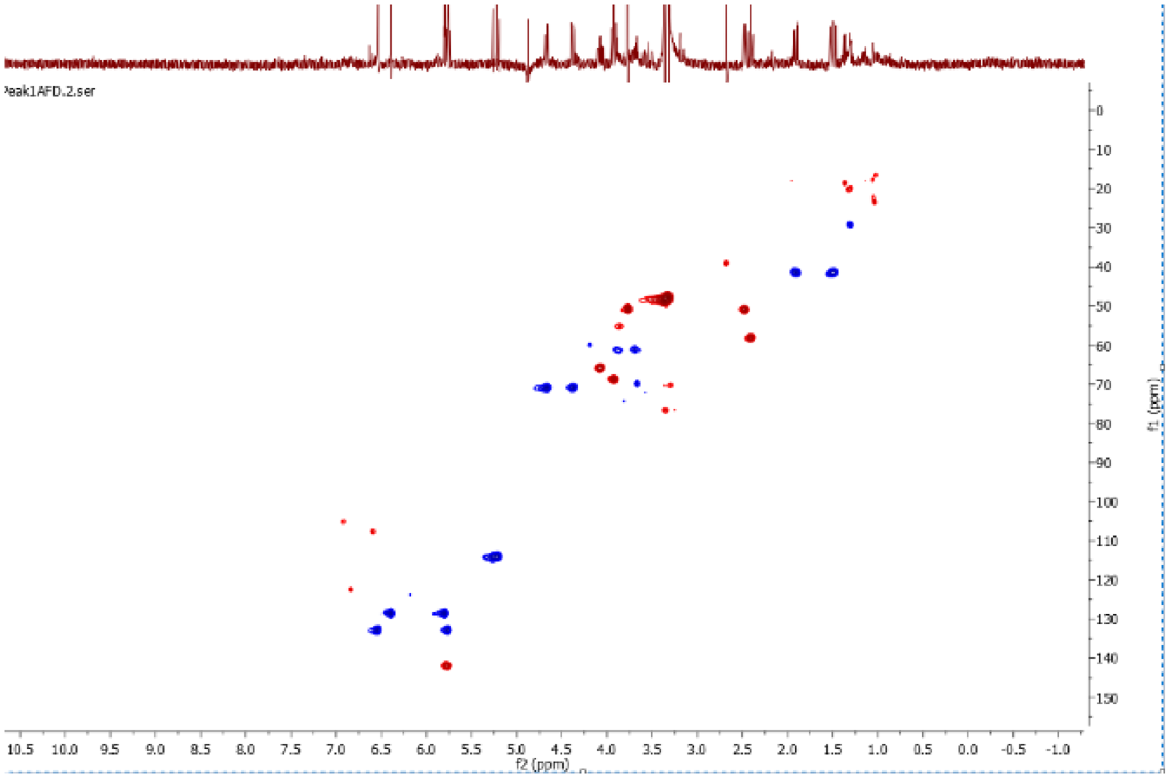

Figure S10: HSQC spectrum of lasiopulide

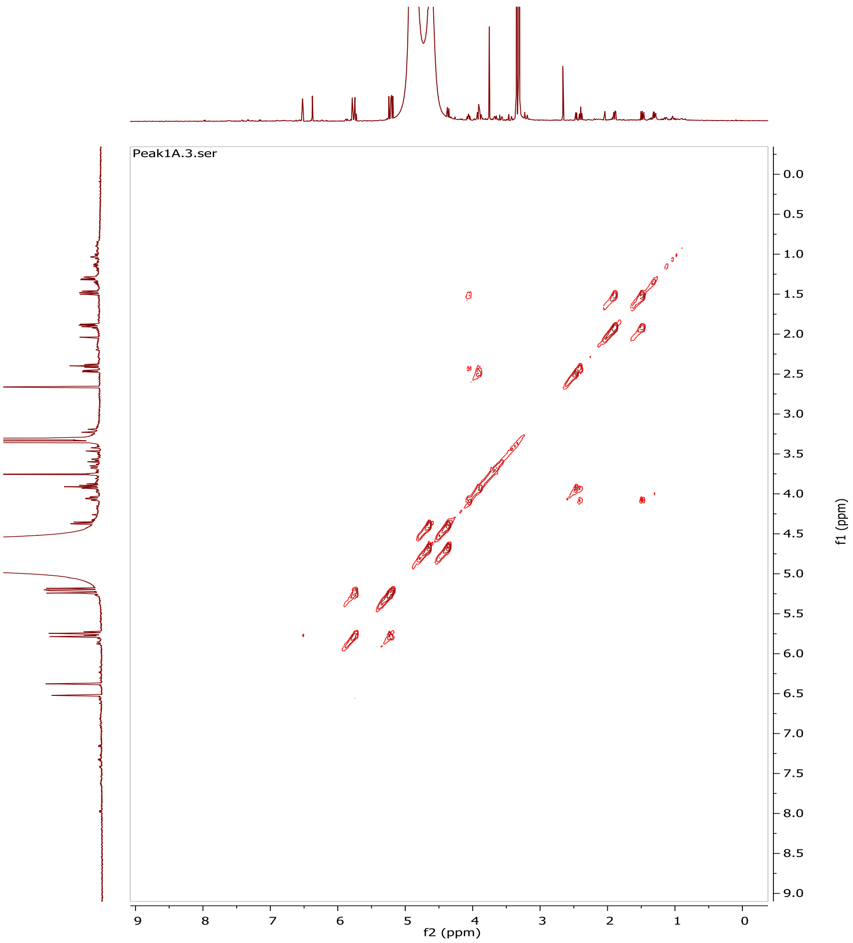

Figure S11: COSY spectral of lasiopulide

Table S3. Compounds identified from VAL and OGL by ultra-high-performance liquid chromatography quadrupole time of flight tandem mass spectrometry UPLC-QTOF-MS

No	Rt	MF	Calc. m/z $[M+H]^+$, $[M-H]^-$	Obs. m/z $[M+H]^+$, $[M-H]^-$	Mass error	MS^E fragmentation	Identification	Source
1	0.8	$C_{13}H_{16}O_8$	299.0764	299.2480	-2.5	178.0632 $[M-H-C_7H_5O_2]^-$, 135.0231 $[M-H-C_6H_{12}O_6]^-$, 89.0347 $[M-H-C_6H_{12}O_6-HCOOH]^-$	Benzoic acid 4-O-*b*-glucoside*	VAL-AB OGL-AB
2	0.9	$C_7H_6O_5$	171.1223	171.1233	0.3	153.1070 $[M+H-H_2O]^+$, 135.0917 $[M+H-2H_2O]^+$, 117.0765 $[M+H-3H_2O]^+$, 73.0764 $[M+H-3H_2O-COOH]^+$	Gallic acid*	VAL-AB OGL-AB
3	1.2	$C_{11}H_{12}O_5$	225.2141	225.2145	0.1	206.0484 $[M+H-H_2O]^+$, 188.0338 $[M+H-2H_2O]^+$, 157.0070 $[M+H-2H_2O-CH_3O]^+$, 125.9739 $[M+H-2H_2O-2CH_3O]^+$	Sinapic acid*	VAL-AB OGL-AB
4	1.3	$C_{18}H_{16}O_8$	360.3094	360.3102	0.3	342.2984 $[M+H-H_2O]^+$, 324.2802 $[M+H-2H_2O]^+$, 306.2656 $[M+H-3H_2O]^+$, 288.2510 $[M+H-4H_2O]^+$, 243.2345 $[M+H-4H_2O-CHO_2]^+$	Rosmarinic acid*	VAL-AB OGL-AB
5	1.4	$C_{16}H_{32}O_3$	256.4188	256.4202	1.5	238.4042 $[M+H-H_2O]^+$, 211.4023 $[M+H-CHO_2]^+$	Palmitic acid*	VAL-AB OGL-AB
6	1.5	$C_{30}H_{48}O_3$	456.6924	456.7091	1.5	438.6778 $[M+H-H_2O]^+$, 420.6632 $[M+H-2H_2O]^+$, 405.6291 $[M+H-2H_2O-CH_3]^+$, 390.5950 $[M+H-2H_2O-2CH_3]^+$	Oleanolic acid*	VAL-AB OGL-AB
7	1.84	$C_{15}H_{12}O_7$	303.0583	303.2393	-3.0	285.0428 $[M-H-H_2O]^-$, 162.0364 $[M-H-C_6H_5O_4]^-$, 152.9591 $[M-H-CH_3-C_8H_8O_2]^-$, 132.0231 $[M-H-H_2O-OCH_3-$	Dihydroquercetin*	VAL-AB VAL-KK OGL-AB OGL-KK

						$C_7H_6O_2]^-$, 130.0135 $[M-H-C_{11}H_9O_2]^-$		
8	2.06	$C_{16}H_{18}O_9$	353.0873	353.2948	0.5	253.1135 $[M-H-3H_2O-HCOOH]^-$, 190.0172 $[M-H-3H_2O-C_6H_5O_2]^-$, 144.0301 $[M-H-H_2O-C_7H_{11}O_6]^-$, 125.0351 $[M-H-H_2O-HCOOH-C_9H_8O_3]^-$	Chlorogenic acid*	VAL-AB OGL-AB
9	2.08	$C_{17}H_{20}O_9$	367.1029	367.3211	1.5	336.0702 $[M-H-OCH_3]^-$, 295.1024 $[M-H-4H_2O]^-$, 243.0591 $[M-H-CH_3-C_6H_5O_2]^-$, 189.0549 $[M-H-CH_3-C_9H_7O_3]^-$, 178.0346 $[M-H-C_8H_{13}O_5]^-$	Methyl-3-caffeoylquinate *^	VAL-KK
10	2.43	$C_9H_8O_4$	179.0335	179.1468	1.2	143.0420 $[M-H-2H_2O]^-$, 133.1433 $[M-H-HCOOH]^-$, 108.0265 $[M-H-C_3H_3O_2]^-$	Caffeic acid*	VAL-AB VAL-KK OGL-AB OGL-KK
11	2.5	$C_{21}H_{20}O_{11}$	449.1047	449.1078	6.9	431.1047 $[M+H-H_2O]^+$, 413.1047 $[M+H-2H_2O]^+$, 287.0530 $[M+H-C_6H_{10}O_5]^+$, 269.0426 $[M+H-C_6H_{10}O_5-H_2O]^+$	Luteolin-7-O-glucoside*	VAL-AB OGL-AB
12	2.5	$C_{21}H_{18}O_{12}$	463.0839	463.0871	7.0	444.9309 $[M+H-H_2O]^+$, 449.1048 $[M+H-CH_2]^+$	Kaempferol-3-glucoronide*	VAL-AB OGL-AB
13	2.6	$C_{17}H_{14}O_6$	314.2851	314.2911	3.1	296.2705 $[M+H-H_2O]^+$, 278.2559 $[M+H-2H_2O]^+$, 260.2413 $[M+H-3H_2O]^+$, 242.2267 $[M+H-4H_2O]^+$, 141.1021 $[M+H-4H_2O-C_8H_5]^+$	Salvigenin*	OGL-AB
14	2.7	$C_{25}H_{24}O_{12}$	517.1310	517.1362	6.0	499.1205 $[M+H-H_2O]^+$, 482.2721 $[M+H-2H_2O]^+$, 466.2251 $[M+H-3OH]^+$	1,3-di-O-caffeoylquinic acid*	VAL-AB
15	2.7	$C_{18}H_{16}O_7$	344.3104	344.3212	1.0	326.2958 $[M+H-H_2O]^+$, 308.2812 $[M+H-2H_2O]^+$, 277.2481 $[M+H-2H_2O-CH_3O]^+$,	Xanthomicrol*	VAL-AB OGL-AB

						246.2150 [M+H-2H_2O-$C_2H_6O_2$]$^+$		
16	2.9	$C_{21}H_{18}O_{11}$	447.0893	447.0922	6.4	269.2257 [M+H-$C_6H_9O_6$]$^+$, 176.1235 [M+H-C12H14O7]$^+$	Apigenin-7-O-glucoronide*	OGL-AB
17	3.0	$C_{20}H_{24}O_8$	393.1520	393.1544	6.1	291.1207 [M+H-$C_4H_6O_3$]$^+$, 273.1103 [M+H-$C_4H_6O_3$-H_20]$^+$, 255.0998 [M+H-$C_4H_6O_3$-2H_20]$^+$, 241.0845 [M+H-$C_4H_6O_3$-2H_20-CH_2]$^+$	Vernodalol*	VAL-AB VAL-KK
18	3.1	$C_{27}H_{30}O_{15}$	593.1517	593.4199	1.1	575.1371 [M-H-H_2O]$^-$, 529.0871 [M-H-H_2O-$C_6H_{12}O_6$]$^-$, 394.1305 [M-H-2H_2O-$C_9H_6O_3$]$^-$	Vicenin-2*	VAL-AB OGL-AB
19	3.2	$C_{18}H_{30}O_2$	277.2165	277.2005	-2.8	182.1254 [M-H-C_7H_{11}]$^-$, 168.1221 [M-H-C_8H_{13}]$^-$, 110.0795 [M-H-$C_{11}H_{17}$-H_2O]$^-$	Linolenic acid*	VAL-AB VAL-KK OGL-AB OGL-KK
20	3.3	$C_{27}H_{30}O_{15}$	593.1516	593.4001	1.1	575.1396 [M-H-H_2O]$^-$, 411.0869 [M-H-H_2O-$C_6H_{12}O_5$]$^-$, 287.0536[M-H-H_2O- $C_6H_{12}O_5$-$C_6H_4O_3$]$^-$, 125.0202 [M-H- $C_6H_{12}O_5$-$C_6H_{12}O_6$-$C_6H_4O_3$]$^-$	Kaempferol-3-rutinoside*	VAL-AB OGL-AB
21	3.5	$C_{35}H_{52}O_{11}$	631.3437	631.3477	6.1	631.3437 [M+H-H_2O]$^+$, 617.3746 [M+H-H_2O-CH_2]$^+$, 603.2756 [M+H-H_2O-C_2H_4]$^+$	Vernonioside A_3*	VAL-AB
22	3.5	$C_{18}H_{22}O_6$	355.1443	355.1489	5.0	317.9794 [M+H-H_2O]$^+$, 287.0525 [M+H-C_2H_8O]$^+$, 275.1371 [$C_2H_4O_2$]$^+$, 265.1055 [M+H-C_4H_6O]$^+$, 251.1259 [M+H-$C_4H_8O_2$]$^+$	Combrestatin*	OGL-AB
23	3.6	$C_{35}H_{52}O_{12}$	665.3495	665.3532	5.5	647.3386 [M+H-H_2O]$^+$, 485 [M+H-$C_6H_{12}O_6$]$^+$, 687.3313 [M+H+Na]$^+$	Vernonioside F*	VAL-AB

24	3.7	$C_{29}H_{42}O_6$	487.3025	487.3054	6.6	487.3022 $[M+H]^+$, 469.2917 $[M+H-H_2O]^+$, 455.3118 $[M+H-H_2O-CH_2]^+$	Vernoniol A4*	VAL-AB
25	3.7	$C_{29}H_{42}O_6$	487.3018	487.3054	5.5	469.2918 $[M+H-H_2O]^+$, 451.1914 $[M+H-2H_2O]^+$, 413.1763 $[M+H-C_3H_6O_2]^+$, 396.1993 $[M+H-H_2O-C_3H_5O_2]^+$	Vernoniol A4*^	OGL-KK
26	4.0	$C_{35}H_{52}O_{11}$	649.3541	649.3582	6.4	631.3437 $[M+H-H_2O]^+$, 617.3646 $[M+H-H_2O-CH_2]^+$, 603.3856 $[M+H-H_2O-C_2H_4]^+$	Vernonioside A4*	VAL-AB
27	4.1	$C_{37}H_{54}O_{12}$	691.3650	691.3688	5.5	649.3545 $[M+H-CH_2CO]^+$, 631.3439 $[M+H-CH_2CO-H_2O]^+$, 575.3542 $[M+H-C_4H_4O_4]^+$	Veramyoside L*	VAL-AB
28	4.1	$C_{45}H_{50}O_9$	735.3542	735.3586	6.0	714.3444 $[M+H-H_2O]^+$, 691.3642 $[M+H-COO]^+$, 631.3438 $[M+H-C_3H_4O_4]^+$, 487.3024 $[M+H-C_6H_8O_4]^+$	Ainsliatriolide A**	VAL-AB
29	4.2	$C_{35}H_{50}O_{11}$	647.3388	647.3426	5.9	615.3492 [M+H-CH3OH]$^+$, 575.2816 $[M+H-C_4H_8O]^+$, 557.3440 $[M+H-C_2H_4O_4]^+$, 471.3175 $[M+H-C_6H_6O_6]^+$	Vernoniamyoside A*	VAL-AB
30	4.3	$C_{35}H_{52}O_{10}$	633.3599	633.3633	5.4	615.3495 $[M+H-H_2O]^+$, 601.3699 $[M+H-CH_3OH]^+$, 489.3181 $[M+H-C_6H_8O_4]^+$, 471.3077 $[M+H-C_6H_{10}O_5]^+$, 453.2971 $[M+H-C_6H_{12}O_6]^+$	Vernonioside B_1*	VAL-AB
31	4.4	$C_{33}H_{48}O_9$	589.3332	589.3379	6.6	571.3229 $[M+H-H_2O]^+$, 473.3302 $[M+H-C_4H_6O_4]^+$, 427.2811{M+H-$C_6H_{10}O_5]^+$, 409.2632 $[M+H-C_6H_{12}O_6]^+$	Piscidinol F*^	VAL-KK
32	4.5	$C_{29}H_{42}O_5$	471.3973	471.3105	6.7	453.2969 $[M+H-H_2O]^+$, 441.3334 [M+H-	Vernoniol B_1*	VAL-AB

						$CH_2O]^+$,409.2709 $[C_2H_6O_2]^+$		
33	4.6	$C_{29}H_{40}O_5$	469.2920	469.2949	6.1	453.2968 $[M+H-H_2O]^+$, 439.3179 $[M+H-CH_2OH]^+$, 425.2655 $[M+H-C_2H_4O]^+$	Vernonioside A_3 Aglycone*^	VAL-KK OGL-KK
34	4.6	$C_{21}H_{20}O_{12}$	463.0866	463.3609	2.3	318.0758 $[M-H-2H_2O-C_6H_5O_2]^-$, 178.0513 $[M-H-C_{15}H_9O_6]^-$, 159.0379 $[M-H-C_6H_{12}O_6-C_6H_4O_3]^-$	Isoquercetin*	VAL-AB VAL-KK OGL-AB OGL-KK
35	4.9	$C_{15}H_{10}O_7$	303.2393	303.0501	2.0	153.1162 $[M+H-C_8H_6O_3]^+$, 151.0212 $[M+H-C_7H_4O_4]^+$, 122.0378 $[M+H-C_9H_6O_4]^+$	Quercetin*	VAL-AB
36	4.9	$C_{27}H_{30}O_{16}$	611.1652	611.4069	2.1	447.1026 $[M+H-C_6H_{12}O_5]^+$, 267.0518 $[M+H-C_6H_{12}O_6-C_6H_{12}O_5]^+$, 158.0287 $[M+H-C_6H_{12}O_6-C_6H_{12}O_5-C_6H_5O_2]^+$, 131.0222 $[M+H-C_6H_{12}O_6-C_6H_{12}O_5-C_7H_4O_3]^+$	Rutin*	VAL-AB VAL-KK OGL-AB OGL-KK
37	5.0	$C_{37}H_{54}O_{11}$	675.3703	675.3739	5.3	661.4277 $[M+H-CH_2]^+$, 647.3749 $[M+H-CH_3CH]^+$, 631.3441 $[M+H-C_2H_4O]^+$, 615.3493 $[M+H-C_2H_4O_2]^+$, 601.3701 $[M+H-C_2H_2O_3]^+$	Vernonioside B_3*	VAL-AB
38	5.1	$C_{38}H_{52}O_{13}$	717.3443	717.3481	5.3	701.3485 $[M+H-H_2O]^+$, 665.3485 $[M+H-2H_2O]^+$, 647.3485 $[M+H-3H_2O]^+$, 603.3251 $[M+H-3H_2O-COOH]^+$	Aleppicatine*^	VAL-KK OGL-KK
39	5.2	$C_{35}H_{52}O_8$	601.3701	601.3735	5.6	573.3760 $[M+H-2CH_2]^+$, 487.3013 $[M+H-C_6H_{10}O_2]^+$, 471.3074 $[M+H-C_6H_{10}O_3]^+$, 453.2971 $[M+H-C_6H_{12}O_4]^+$, 439.3179 $[M+H-C_6H_{10}O_5]^+$	Cimicinol*	OGL-AB
40	5.3	$C_{35}H_{50}O_6$	567.3681	567.3680	0.2	549.3863 $[M+H-H_2O]^+$, 511.3363	Hyperfol*	OGL-AB

						$[M+H-C_3H_4O]^+$, 471.3084 $[M+H-C_2H_8O_4]^+$, 453.3335 $[M+H-C_6H_{10}O_2]^+$, 435.3235 $[M+H-C_6H_{12}O_3]^+$, 407.3291 $[M+H-C_6H_8O_5]^+$		
41	5.3	$C_{35}H_{52}O_9$	617.3646	617.3684	6.1	599.3538 $[M+H-H_2O]^+$, 528.3697 $[M+H-C_2H_2O_4]^+$, 486.2154 $[M+H-OH-C_6H_9O_2]^+$, 471.3070 $[M+H-OH-C_6H_9O_2-CH_3]^+$, 456.3161 30702 $[M+H-OH-C_6H_9O_2-CH_3CH_3]^+$	Veramyoside K*	VAL-AB
42	5.5	$C_{29}H_{42}O_4$	455.3126	455.3156	6.5	437.3021 $[M+H-H_2O]^+$, 409.2707 $[M+H-C_2H_6O]^+$, 395.2914 $[M+H-C_2H_4O_2]^+$,	Reaxys ID: 23094383*	VAL-KK OGL-KK
43	5.6	$C_{30}H_{48}O_5$	489.3570	489.3575	1.0	471.3461 $[M+H-H_2O]^+$, 453.3360 $[M+H-2H_2O]^+$, 435.3260 $[M+H-3H_2O]^+$, 425.3410 $[M+H-C_2H_8O_2]^+$, 407.3302 $[M+H-C_2H_8O_2- H_2O]^+$, 391.3328 $[M+H-C_2H_{10}O_4]^+$	Tormentic acid*	OGL-AB
44	5.7	$C_{37}H_{52}O_9$	641.3627	641.3684	8.8	623.3536 $[M+H-H_2O]^+$, 608.3192 $[M+H- H_2O-CH_3]^+$, 593.2848 $[M+H-H_2O- C_2H_6]^+$, 471.3078 $[M+H-C_7H_5O_4-CH_3]^+$	Galloylarjunolic acid*^	VAL-KK
45	5.8	$C_{40}H_{52}O_9$	677.3697	677.3694	-1.9	659.3595 $[M+H-H_2O]^+$, 641.3623 $[M+H-2H_2O]^+$, 607.3253 $[M+H-C_4H_6O]^+$, 593.2374 $[M+H-C_4H_4O_2]^+$, 515.3182 $[M+H-C_6H_{10}O_5]^+$, 497.3090 $[M+H-C_6H_{12}O_6]^+$	Bislangduoid*	OGL-AB
46	5.8	$C_{21}H_{36}O_4$	356.2666	356.2686	5.9	338.2538 $[M+H-H_2O]^+$, 323.2194 [M+H- H2O-	Tomentogenin**	VAL-AB

						$CH_3OH]^+$, 308.1850 $[M+H- H_2O-C_2H_6]^+$		
47	5.9	$C_{16}H_{20}O_6$	310.1352	310.1394	0.4	291.1342 $[M+H-H_2O]^+$, 247.1342 $[M+H-OCH_3]^+$	Lasiopulide*	VAL-KK
48	6.4	$C_{21}H_{36}O_4$	353.2661	353.2686	7.1	293.2085 $[M+H-C_2H_4O_2]^+$, 261.2190 $[M+H-C_2H_4O_4]^+$, 239.9555 $[M+H-C_6H_{10}O_2]^+$	Diterpene*	OGL-AB
49	6.9	$C_{30}H_{48}O_4$	473.3624	473.3625	0.2	455.3514 $[M+H-H_2O]^+$, 437.3412 $[M+H-2H_2O]^+$, 409.3463 $[M+H-C_2H_8O_2]^+$, 391.3355 $[M+H-C_2H_8O_2-H_2O]^+$, 355.2844 $[M+H-C_6H_{14}O_2]^+$	Maslinic acid*	OGL-AB
50	7.0	$C_{40}H_{56}O_8$	665.4047	655.4048	0.1	647.3945 $[M+H-H_2O]^+$, 635.2817 $[M+H-CHO]^+$, 601.2643 $[M+H-C_2H_8O_2]^+$, 551.3761 $[M+H-C_6H_{10}O_2]^+$	Triterpenoid compound*	OGL-AB<br>OGL-KK
51	7.1	$C_{22}H_{40}O_5$	385.2949	385.2958	0.2	367.2841 $[M+H-H_2O]^+$, 352.8982 $[M+H-CH_3OH]^+$, 326.2009 $[M+H-C_2H_4O_2]^+$, 311.0081 $[M+H-C_3H_6O_2]^+$	Santinol*	OGL-AB
52	7.3	$C_{28}H_{32}O_8$	497.2171	497.2179	-0.1	467.2070 $[M+H-CHO]^+$, 366.9352 $[M+H-C_6H_{11}O_3]^+$, 315.0860 $[M+H-C_6H_{14}O_6]^+$	Oxogedunin*	OGL-AB
53	7.4	$C_{31}H_{38}O_6$	555.3505	555.3528	4.0	538.3427 $[M+H-H_2O]^+$, 523.2459 $[M+H-CH_3OH]^+$, 513.3403 $[M+H-CH_2CO]^+$, 393.3122 $[M+H-C_6H_{10}O_5]^+$,	Amoritin*^	VAL-KK
54	7.8	$C_{28}H_{44}O_{11}$	557..2957	557.2956	-0.1	539.2852 $[M+H-H_2O]^+$, 487.3570 $[M+H-C_4H_6O]^+$, 471.3073 $[M+H-C_4H_6O_2]^+$, 443.3319 $[M+H-C_6H_{10}O_2]^+$, 427.2186 $[M+H-C_6H_8O_2-H_20]^+$, 413.2637 $[M+H-C_6H_8O_2-CH_2]^+$	Viteoside A*	OGL-AB
55	7.9	$C_{30}H_{54}O_9$	559.3836	559.3841	0.9	540.4470 $[M+H-H_2O]^+$, 501.3761	No hits**^**	VAL-KK

						[M+H-CH_2COO]$^+$, 413.3254 [M+H-$C_6H_{10}O_4$]$^+$, 391.3416 [M+H-$C_8H_8O_4$]$^+$		
56	7.9	$C_{25}H_{30}O_4$	395.2195	395.2217	5.4	363.2297 [M+H-CH_3OH]$^+$, 325.2115 [M+H-C_4H_6O]$^+$, 283.1746 [M+H-$C_6H_8O_2$]$^+$, 201.0895 [M+H-$C_6H_{12}O_6$-CH_2]$^+$	Kazinol *^	VAL-KK
57	8.1	$C_{34}H_{40}O_{10}$	609.2718	609.2694	3.9	549.4886 [M+H-$C_2H_4O_2$]$^+$, 513.3404 [$C_2H_8O_4$]$^+$, 447.2878 [M+H-$C_6H_{10}O_5$]$^+$	Limonoid*^	VAL-KK OGL-KK
58	8.3	$C_{30}H_{46}O_2$	439.3541	439.3571	6.7	411.3594 [M+H-2CH_2]$^+$, 392.3133 [M+H-C_2H_7O]$^+$, 369.2210 [M+H-C_4H_6O]$^+$, 325.2123 [M+H-$C_6H_{10}O_2$]$^+$, 279.1586 [M+H-$C_6H_8O_5$]$^+$, 261.2196 [M+H-$C_6H_{10}O_6$]$^+$	Salviniol*	OGL-AB OGL-KK
59	8.6	$C_{34}H_{40}O_{11}$	625.2619	625.2643	3.9	607.2508 591.2523 [M+H-H_2O]$^+$, 593.5099 [M+H-CH_2OH]$^+$	Tetranotreterpenes*	VAL-AB VAL-KK OGL-AB OGL-KK
60	9.0	$C_{34}H_{40}O_9$	593.2715	593.2745	5.8	547.3558 [M+H-C_2H_6O]$^+$, 537.3877 [M+H-C_3H_4O]$^+$, 531.2662 [M+H-$C_2H_6O_2$]$^+$, 521.3904 [M+H-$C_3H_4O_2$]$^+$, 515.3865 [M+H-$C_3H_{10}O_2$]$^+$	Moreollic acid*	OGL-AB OGL-KK
61	9.2	$C_{32}H_{40}O_8$	553.2779	553.2796	3.0	535.2670 [M+H-H_2O]$^+$, 505.2504 [M+H-C_2H_8O]$^+$,481.3620 [M+H-$C_3H_4O_2$]$^+$, 455.2141 [M+H-$C_2H_{10}O_4$]$^+$, 406.3268 [M+H-$C_6H_{11}O_4$]$^+$	Khayasin*^	OGL-KK
62	9.3	$C_{32}H_{38}O_7$	535.2671	535.2690	3.7	505.2507 [M+H-CHO]$^+$, 493.1180 [M+H-CH_3CO]$^+$, 465.2588 [M+H-C_4H_6O]$^+$, 455.2144 [M+H-$C_3H_4O_2$]$^+$,	Oblongifolin *^	OGL-KK

						441.3695 [M+H-$C_2H_6O_4$]$^+$, 406.3267 [M+H-$C_6H_9O_3$]$^+$		
63	9.5	$C_{35}H_{42}O_{10}$	623.2826	623.2851	4.0	578.2815 [M+H-C_2H_5O]$^+$, 573.3964 [M+H-CH_3OH-H_2O]$^+$, 562.5630 [M+H-$C_2H_5O_2$]$^+$, 553.2790 [M+H-C_4H_6O]$^+$, 546.8273 [M+H-$C_3H_9O_2$]$^+$, 523.3569 [M+H-$C_5H_8O_2$]$^+$, 505.2502 [M+H-$C_6H_{14}O_2$]$^+$, 463.2781 [M+H-$C_6H_8O_5$]$^+$	Taxuspine*	OGL-AB OGL-KK
64	9.8	$C_{35}H_{42}O_9$	607.2886	607.2902	2.6	561.3723 [M+H-C_2H_6O]$^+$, 515.4096 [M+H-$C_3H_8O_3$]$^+$, 505.2502 [M+H-$C_5H_{10}O_2$]$^+$, 497.3563 [M+H-$C_6H_6O_2$]$^+$, 469.2688 [M+H-$C_4H_{10}O_5$]$^+$	Taxinine*	OGL-AB OGL-KK
65	10.0	$C_{36}H_{44}O_{11}$	653.2927	653.2956	4.5	621.3030 [M+H-CH_3OH]$^+$, 607.3875 [M+H-C_2H_6O]$^+$, 595.4681 [M+H-$C_2H_2O_2$]$^+$, 582.6963 [M+H-C_4H_7O]$^+$, 549.2821 [M+H-$C_5H_{12}O_2$]$^+$, 538.6705 [M+H-$C_6H_{11}O_2$]	Moluccensin*	OGL-AB OGL-KK
66	10.1	C10H12O2	163.0759	163.1908	-2.2	122.0453 [M-H-C_3H_5]$^-$, 105.0495 [M-H-OCH_3-C_2H_3]$^-$	Eugenol*	OGL-AB
67	11.0	$C_{29}H_{48}O_2$	429.3717	429.3727	2.5	398.3407 [M+H-CH_3OH]$^+$, 362.9252 [M+H-C_4H_4O]$^+$, 352.8965 [M+H-$C_3H_{10}O_2$]$^+$, 294.9381 [M+H-$C_6H_{16}O_3$]$^+$	Saringosterol*	OGL-AB OGL-KK
68	12.8	$C_{19}H_{22}O_7$	363.9262	363.9274	3.3	345.1300 [M+H-H_2O]$^+$, 276.1025 [M+H-H_2O-C_4H_5O]$^+$, 262.0812 [M+H- H_2O-C_4H_5O-CH_2]$^+$	Vernolide*	VAL-AB

69	12.9	$C_{21}H_{38}O_4$	354.2757	354.2770	-3.3	399.2739 [M-H-COO]$^-$, 324.2188 [M-H-C_2H_5]$^-$, 238.1477 [M-H-H_2O-C_7H_{13}]$^-$, 202.1102 [M-H-$C_{11}H_{19}$]$^-$, 151.1162 [M-H-C_8H_{15} - $C_3H_7O_3$]$^-$	2-Monolinolein*	VAL-KK OGL-KK
70	13.0	$C_{18}H_{34}O_2$	282.2558	282.2559	-0.1	283.2731 [M+H]$^+$, 97.1020 [M+H-C_5H_{11} - $C_6H_{11}O_2$]$^+$, 86.1034 [M+H - $C_{12}H_{21}O_2$]$^+$, 72.0877 [M+H- $C_{13}H_{23}O_2$]$^+$	Oleic acid*	VAL-AB VAL-KK OGL-AB OGL-KK

Rt—Retention time; MF—Molecular formula; AB—Abraka; KK—Kokori; VAL—*Vernonia amygdalina* leaves; OGL—*Ocimum gratissimum* leaves

*Previously discovered in plants; *^Only discovered in crude oil species
